# Corrigendum

**DOI:** 10.1111/iwj.14364

**Published:** 2023-09-05

Corrigendum to Synergetic integrations of bone marrow stem cells and transforming growth factor‐β1 loaded chitosan nanoparticles blended silk fibroin injectable hydrogel to enhance repair and regeneration potential in articular cartilage tissue.

Zheng D, Chen T, Han L, et al. Synergetic integrations of bone marrow stem cells and transforming growth factor‐β1 loaded chitosan nanoparticles blended silk fibroin injectable hydrogel to enhance repair and regeneration potential in articular cartilage tissue. Int Wound J. 2022;19(5):1023–1038. doi:10.1111/iwj.13699

Figure 3 is in low resolution and the images are magnified. Below is the corrected Figure 3 with high‐resolution images.

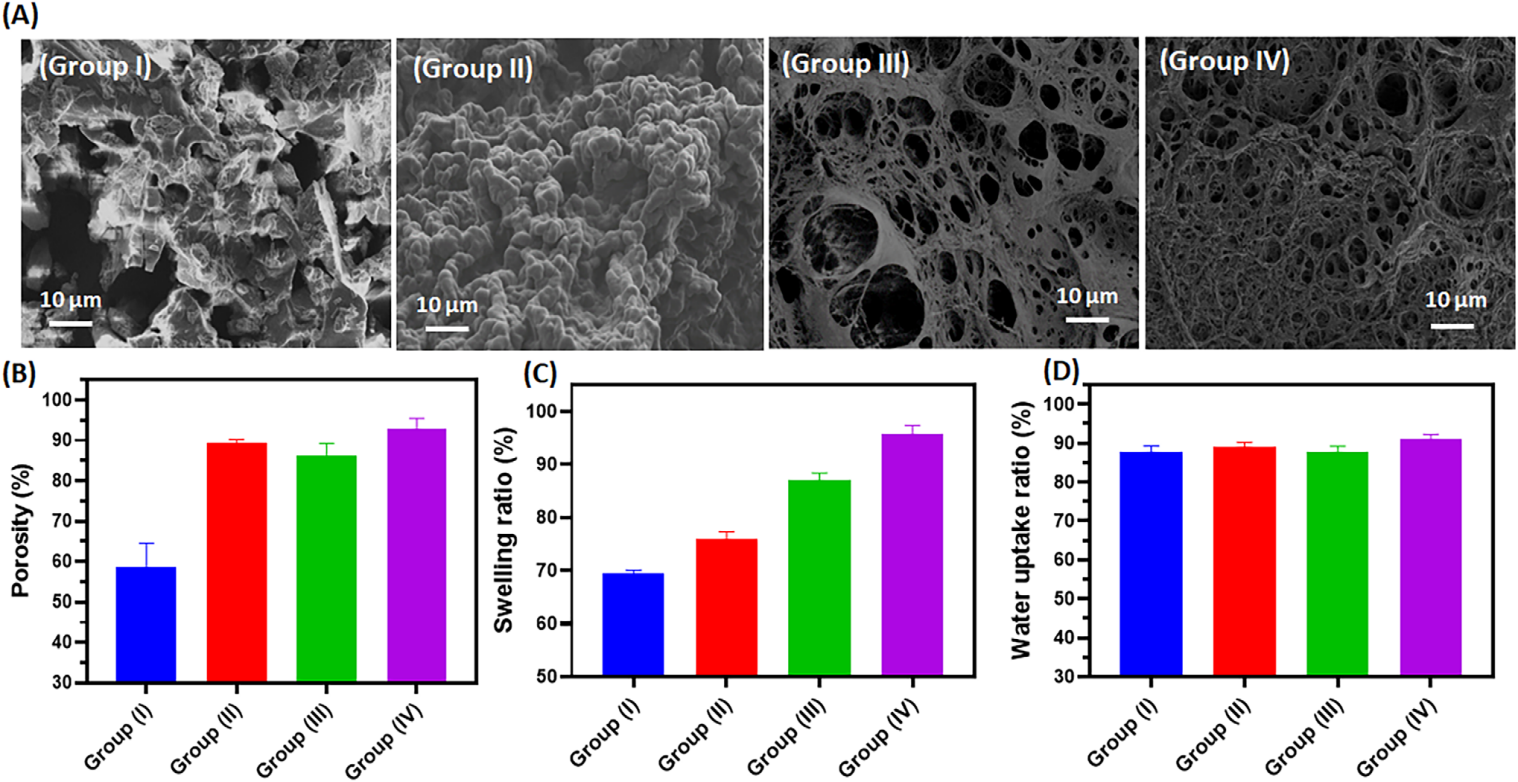

We apologize for this error.

